# The association between health workforce availability and HIV-program outcomes in Côte d’Ivoire

**DOI:** 10.1186/s12960-022-00715-2

**Published:** 2022-02-19

**Authors:** Derick Akompab Akoku, Kirkby D. Tickell, Kouadio R. Niamien, Kathryn E. Kemper, Doumbia Yacouba, Seydou Kouyate, Daniel A. Kouassi, Shirish Balachandra, Meghan Swor, Audrey Knutson Luxenberg, Steve Gloyd, Ahoua Kone

**Affiliations:** 1 Health Alliance International, Abidjan, Côte d’Ivoire; 2 Department of Global Health, University of Washington, Seattle, USA; 3 Health Alliance International, Seattle, WA USA; 4 Ministère de la Santé et de l’Hygiène Publique, Abidjan, Côte d’Ivoire; 5 Affiliation of Center for Global Health/Division of Global HIV and TB (DGHT), Centers for Disease Control and Prevention, Abidjan, Côte d’Ivoire; 6 Center for Global Health/Division of Global HIV and TB (DGHT), Centers for Disease Control and Prevention, Abidjan, Côte d’Ivoire

**Keywords:** HIV/AIDS, Human resources for health, Health workforce allocation

## Abstract

**Objective:**

The purpose of this study was to assess the distribution of HIV-program staff and the extent to which their availability influences HIV programmatic and patient outcomes.

**Methods:**

The study was a facility level cross-sectional survey. Data from October 2018 to September 2019 were abstracted from HIV program reports conducted in 18 districts of Côte d’Ivoire. The distribution of staff in clinical, laboratory, pharmacy, management, lay, and support cadres were described across high and low antiretroviral therapy (ART) volume facilities. Non-parametric regression was used to estimate the effects of cadre categories on the number of new HIV cases identified, the number of cases initiated on ART, and the proportion of patients achieving viral load suppression.

**Results:**

Data from 49,871 patients treated at 216 health facilities were included. Low ART volume facilities had a median of 8.1 staff-per-100 ART patients, significantly higher than the 4.4 staff-per-100 ART patients at high-ART volume facilities. One additional laboratory staff member was associated with 4.30 (IQR: 2.00–7.48, *p* < 0.001) more HIV cases identified and 3.81 (interquartile range [IQR]: 1.44–6.94, *p* < 0.001) additional cases initiated on ART. Similarly, one additional lay worker was associated with 2.33 (IQR: 1.00–3.43, *p* < 0.001) new cases identified and 2.24 (IQR: 1.00–3.31, *p* < 0.001) new cases initiated on ART. No cadres were associated with viral suppression.

**Conclusions:**

HCWs in the laboratory and lay cadre categories were associated with an increase in HIV-positive case identification and initiation on ART. Our findings suggest that allocation of HCWs across health facilities should take into consideration the ART patient volume. Overall, increasing investment in health workforce is critical to achieve national HIV goals and reaching HIV epidemic control.

**Supplementary Information:**

The online version contains supplementary material available at 10.1186/s12960-022-00715-2.

## Background

HIV and AIDS still represent a significant global health burden with an estimated 38 million individuals living with HIV worldwide, and 25.4 million accessing antiretroviral therapy (ART) [1]. In many countries in sub-Saharan Africa (SSA), the increase in the number of ART patients poses a huge challenge to health systems [2]. The Joint United Nations Programme on HIV/AIDS (UNAIDS) Fast-Track strategy to end the AIDS epidemic by 2030 identifies three critical outcomes by calling for 95% of all people living with HIV to know their status, 95% of people who know their status to be on treatment, and 95% of people on treatment to have a suppressed viral load [3]. The shortage of human resources in the health sector in SSA has been identified as a key barrier to strengthening the health system and achieving the UNAIDS goals [4]. Many countries with the highest numbers of HIV cases have inadequate numbers of qualified healthcare workers(HCWs) to provide comprehensive care to patients [5].

In some countries, less than 52% of the approved HRH positions are filled due to HCW shortages, and there is often a maldistribution of HCWs across health facilities. These staffing levels are inadequate to meet the growing needs of patients on ART [6].

The U.S. President’s Emergency Plan for AIDS Relief (PEPFAR) and other donors have invested significant funds to support governments in SSA to strengthen human resources for health. This investment has created opportunities to optimize ART service delivery through differentiated models of care that reduce overcrowding, and improve service quality and efficiency [7–9]. In Malawi, PEPFAR supported the recruitment, training, and deployment of HCWs across health facilities to support HIV services delivery. An evaluation of this program showed that the intervention resulted in improved availability, utilization, and quality of HIV/AIDS services and HIV outcomes [6].

In Cote d’Ivoire, there is very limited data on the distribution and effectiveness of human resources for health and HIV-related outputs and outcomes. The absence of such data makes it difficult for policy makers to understand how to target further investments or redistribute available human resources, optimize health worker allocation and achieve HIV epidemic control. In addition, the available human resources for health literature has focused on correlating county-, regional- or country-level cadre availability data with population level health outcome data [10–13]. Data aggregated to administrative region or country-level is vulnerable to ecological bias, where the observed associations cannot be replicated at the facility or patient level and very few published studies have specifically focused on the effects of PEPFAR support staff on the UNAIDS 95–95–95 goals.

The objectives of this evaluation were to assess the distribution of different categories of HCWs in a large PEPFAR funded HIV prevention, care and treatment program and estimate the association between HCW availability at health facilities and HIV program outputs related to the three critical UNAIDS 95–95–95 goals. The findings of this evaluation may guide the Ministry of Health and PEPFAR implementing partners in making appropriate decisions related to staffing investments in health facilities across the country.

## Methods

### Study design, selection of health facilities and setting

**Fig. 1:**
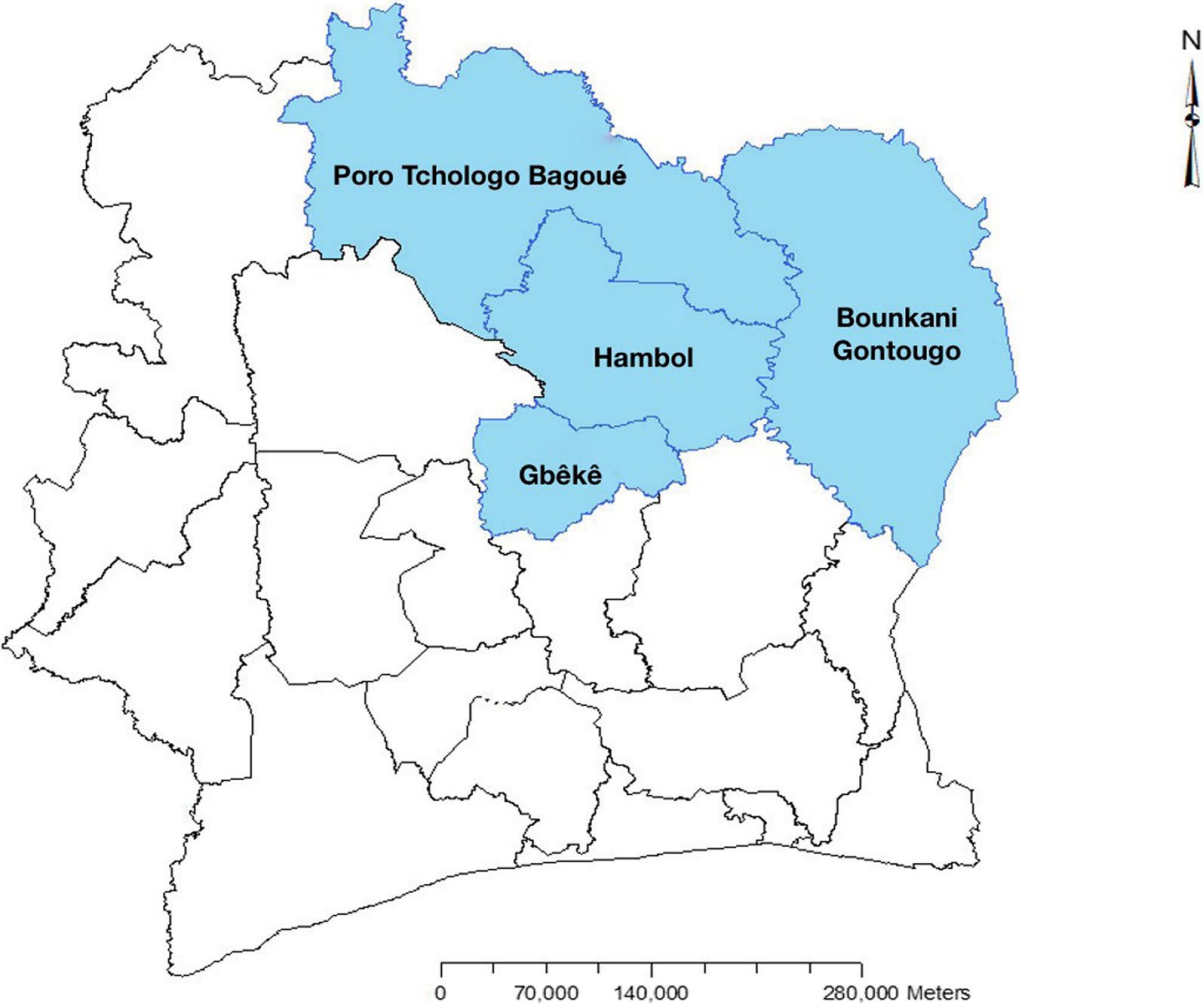
Côte d’Ivoire Health regions served by Health Alliance International and the Ministry of Health

We evaluated the relationship between site level human resources and HIV program outcomes between October 2018 and September 2019 at health facilities supported by Health Alliance International (HAI) in Côte d’Ivoire. HAI is an international US based non-profit organization which, with funding from the U.S Centers for Disease Control and Prevention (CDC) through PEPFAR, has been collaborating since 2007 with the Ministry of Health in Côte d’Ivoire to implement HIV prevention, care and treatment services. In 2019, HAI-supported health facilities across 18 health districts in four regions (Fig. 1). All HAI-supported facilities providing HIV testing, care and treatment services were eligible for inclusion in this analysis. Health facilities with less than 25 patients receiving ART were excluded, in accordance with Côte d’Ivoire’s national definition of an HIV treatment facility.

Côte d’Ivoire has an estimated population of 22.6 million and the HIV prevalence is estimated to be between 2.9 and 3.7% among 15–49 years [14, 15]. By the end of 2018, it was estimated that 458,434 people were living with HIV in the country and the number of people on ART had increased substantially from 4536 in 2004 to 228,174 in 2017 [16].

### Outcome variables

We evaluated three outcomes: the number of individuals newly diagnosed as HIV-positive who received their test results during the evaluation period, the number of HIV-positive individuals who newly initiated ART during this time period, and the percentage of ART patients with a suppressed viral load result (< 1000 copies/ml) within the past 12 months. The percentage of ART patients whose viral load was suppressed was calculated among ART patients who had a documented viral load result within the past 12 months.

The primary source of data was HIV management registers for testing and treatment at each site. Data were abstracted by a trained evaluation team and included site-level data on the number of individuals who tested HIV-positive, who were initiated on treatment, who received a viral load test, and were virally suppressed. Data were cross-checked against health facility assessment reports, HIV testing registers, HIV treatment registers, ART dispensation registers (pharmacy records), patient medical charts and electronic medical records. Only individuals who tested HIV-positive in the health facility and initiated on ART were included in the analysis. Those who tested HIV-positive in the community and referred for treatment in the health facility were excluded as we intended to estimate the true linkage rate (i.e., the proportion of individuals who tested HIV-positive in the facility and initiated on ART) which is a key measure used in PEPFAR-funded projects to determine the performance of health facilities in initiating HIV-positive cases diagnosed at the health facilities on ART.

Human resource data capturing Ministry of Health and PEPFAR staffing during the evaluation period were collected from each site in September 2019. We considered the six main categories of HCWs that are widely used in PEPFAR-funded projects [8]. The six included categories of HCWs were clinical, pharmacy, laboratory, management, lay workers, and support staff (Additional file 1: Table S1). Only HCWs involved in HIV-related activities were considered in the evaluation. HCWs involved in non-HIV activities (e.g., malaria, family planning etc.) were not considered.

### Ethical consideration

The project was reviewed in accordance with CDC human research protection procedures and was determined to be research, but CDC investigators did not interact with human subjects or have access to identifiable data or specimens for research purposes. Data were collected from routine program records to evaluate and guide human resources planning and investment. The data were de-identified aggregated data from HAI-supported health facilities, and ethical approval was not necessary. However, the Ministry of Health of Côte d’Ivoire granted permission to conduct the evaluation and collaborated with data collection.

### Data analysis

Data were entered and cleaned in an Excel database and then analyzed in STATA version 14.2 (STATA Corp., College Station, TX). To better describe the data, health facilities were categorized into high and low ART patient volume sites using a definition employed by PEPFAR in Côte d’Ivoire during Fiscal Year 2020 (October 1st, 2019 to September 30th, 2020) [17]. Health facilities, were ranked in descending order, by the total number of patients receiving ART at that facility at the end of September 2019. The sites that captured 70% of the cumulative total of ART patients across the health facilities in this evaluation were considered high volume, while sites that captured the remaining 30% of ART patients were considered low volume. The distribution of the independent variables was assessed using medians with inter-quartile ranges (IQRs) for continuous variables and frequencies with percentages for categorical variables. Crude differences in the outcome variables between high and ART patient volume sites were tested using Wilcoxon rank-sum test.

We calculated the number of HCWs per 100 ART patients by dividing the number of available staff providing HIV-related services in each HCW category by the number of individuals alive and receiving treatment at that facility. Kernel-based regularized least squares regression models were used to examine the relationship between the number of staff in each HCW category and the three HIV-program outcomes consisting of the number of new cases identified, the number of patients initiated on ART, and the percentage of patients achieving viral load suppression. Kernel-based regularized least squares regression (Stata “krls” package) is a non-parametric approach which was used, because the assumptions required to perform a parametric test were not met [18, 19]. Crude models included all six cadre categories (clinical, pharmacy, laboratory, management, lay workers, and support staff) and a continuous variable for the total number of ART patients receiving treatment at the facility. We also constructed adjusted models which included potential site level confounders: region (dummy variables), facility type (publicly owned vs other) and facility level, where primary refers to all rural facilities, secondary refers to urban facilities designated, and tertiary refers to general hospitals and specialized health facilities. The association between HCW categories and the three outcomes were tested for non-linearity in adjusted models by inclusion of quadratic terms. Non-linear terms were retained if the *p* value for the squared HCW category was less than 0.10.

## Results

Of 229 eligible health facilities, 13 (5.7%) treated fewer than 25 patients. These health facilities were excluded from this study, resulting in 216 sites retained. Collectively, by the end of September 2019, 49,871 patients were receiving ART across these sites. Fifty-eight (26.9%) of these sites were high ART patient volume sites, while 158 (73.1%) were low ART patient volume sites (Table 1). The median number of patients receiving treatment at high volume sites was 436 (IQR: 323–700), while at low-volume sites it was 76 (IQR: 44–123). High and low volume sites were evenly distributed across all four regions. Of these, 197 sites (91.2%) were public, 76 (35.2%) were primary, 78 (36.1%) secondary, and 62 (28.7%) were tertiary-level facilities.

**Table 1:** Characteristics of the included health facilities by ART patient volume

	Total	Health facility by ART patient volume	
		High	Low
	*N* (%)	*N* (%)	*N* (%)
Number of health facilities	216 (100)	58 (26.9)	158 (73.1)
Number of patients alive and receiving ART	49,871 (100)	34,894 (70.0)	14,977 (30%)
Health facility designation^a^			
Primary	76 (35.2)	2 (3.5)	74 (46.8)
Secondary	78 (36.1)	23 (39.7)	55 (34.8)
Tertiary	62 (28.7)	33 (56.9)	29 (18.4)
Type of Facility			
Public	197 (91.2)	49 (84.5)	148 (93.7)
FBO/NGO^b^	19 (8.8)	9 (15.5)	10 (6.3)
Health Region			
Bounkani-Gontougo	52 (24.1)	14 (24.0)	38 (24.0)
Gbêkê	74 (34.3)	20 (34.5)	54 (34.2)
Hambol	21 (9.7)	6 (10.3)	15 (9.5)
Poro-Tchologo-Bagoué	69 (31.9)	18 (31.0)	51 (32.3)

^a^Designation: Primary = refers to all facilities with the designation as “Rural”; Secondary = refers to facilities designated as Urban and Tertiary refers to general hospitals and specialized health facilities

^b^FBO/NGO = Faith-based organizations and non-governmental organizations

### Distribution of HCWs

**Fig. 2:**
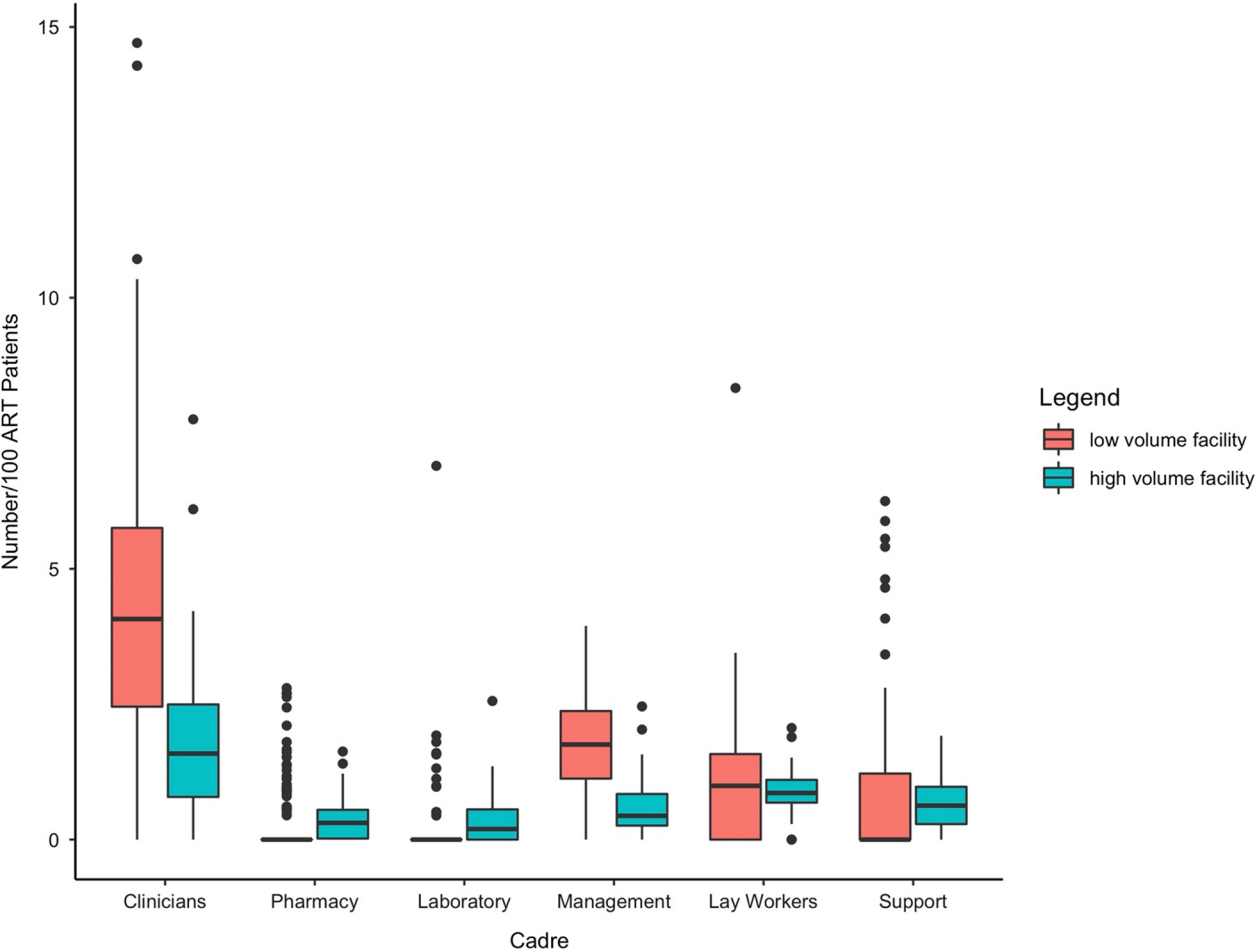
Estimated number of healthcare workers per 100 ART patient

Of the 2577 HCWs providing HIV-related services in this study, 1477 (57.3%) worked at high volume health facilities (Additional file 1: Fig. S1). High volume facilities had a median of 4.4 HCWs (IQR: 3.1–5.7) per 100 ART patients compared to 8.1 HCWs (IQR: 6.1–10.0) per 100 ART patients at low volume sites. However, high volume sites had more staff in three categories of cadres, with a median of 0.31 (IQR: 0.00–0.58) pharmacists per 100 ART patients (Fig. 2), in comparison to 0.00 (IQR: 0.00–0.00) pharmacists at low volume sites. Similarly, higher volume sites had a median of 0.20 (IQR: 0.00–0.57) laboratory and 0.63 (IQR: 0.29–0.98) support staff per 100 ART patients, while low-volume sites had 0.00 (IQR: 0.00–0.00) in both categories. Conversely, low volume sites had a considerably higher median number of clinicians, 4.07 (IQR: 2.44–5.77) vs 1.59 (IQR:0.78–2.53); managers 1.75 (IQR: 0.78–2.53) vs 0.44 (IQR: 0.26–0.87); and slightly more lay workers, 0.99 (IQR: 0.00–1.60) vs 0.86 (IQR: 0.68–1.10), per 100 ART patients than the higher volume sites.

### HIV-program outcomes during the evaluation period

During the evaluation period, 7627 new HIV cases were diagnosed, 4745 (62.2%) of which were in high ART patient volume health facilities (Table 2). There was a significant difference in the median number of HIV-positive cases identified at high patient volume sites (median: 66.0, IQR: 43.0–97.0) compared to low patient volume sites (median: 15.0, IQR: 9.0–24.0, Wilcoxon rank-sum: *p* < 0.001). Of those diagnosed, 7479 (98.1%) were initiated on ART, 61.9% of whom started treatment at high volume health facilities.

**Table 2:** HIV-program outcomes between October 2018 and September 2019

	Total	Health facility ART patient volume				P-value^c^
		High		Low		
	*N*	*N*	(%)	*N*	(%)	
HIV-positive cases diagnosed	7627	4745	(62.2)	2882	(37.8)	** < 0.001**
HIV-positive cases newly initiated on ART^a^	7479	4633	(61.9)	2846	(38.1)	** < 0.001**
Proportion of ART patients with VL suppression^b^	77.9%	80.1%	–	78.3%	–	**0.07**

^a^The true linkage rate was 98.1% (those diagnosed HIV-positive in a facility and initiated on ART) which is a measure of the Test and Treat Policy under HIV/AIDS programs

^b^We computed this indicator by dividing the sum of number of ART patients with suppressed VL results (< 1000 copies/ml) by the sum of the number of ART patients who had a documented VL result within the past 12 months

^c^Wilcoxon rank-sum tests, comparing high and low volume facilities for the variable on that row

There was a significant difference in the median number of HIV-positive cases initiated on ART between high volume health facilities (median: 64.5, IQR: 43–94) and low volume facilities (median: 15.0, IQR: 9.0–23.0, Wilcoxon rank-sum: *p* < 0.001). However, there was no significant difference in the proportion of newly identified cases who were initiated on ART between the high (median: 100.0%, IQR: 99.0–100.0%) and low volume sites (median: 100.0%, IQR: 100.0–100.0%, *p* = 0.569). Overall, the viral suppression rate was 77.9%, with a median of 80.2% (IQR: 0.77–0.85) of patient’s achieving viral suppression in high volume health facilities and 78.3% (IQR: 0.71–0.84; *p* = 0.07) achieving suppression at low-volume sites.

### Association between HCWs availability and program results

In crude models, the majority of the variation in the number of new HIV cases identified (*R*^2^: 0.924), and the number of cases initiated on ART during the evaluation period (*R*^2^: 0.922) was explained by the six cadre categories and the number of ART patient at the beginning of the study period. Addition of the a-priori identified adjustment variables: region, health system level and type of facility; and inclusion of non-linear terms where appropriate led to a modest increase in the proportion of variability explained by the covariates (*R*^2^: 0.959 & *R*^2^: 0.957, respectively).

The crude models (Table 3) found the laboratory cadre to be significantly associated with the number of new HIV patients diagnosed and the number of HIV-positive cases started on ART. No other cadre categories were associated with these outcomes in the crude model. After adjusting for region, health system level and type of facility, an increase of one laboratory staff member was associated with a median of 3.84 (IQR: 2.00–7.48, *p* < 0.001) additional HIV cases identified each year and 3.60 (IQR: 1.74–6.74, *p* < 0.001) more patients initiated on ART. In these adjusted models, an increase of one lay health worker was also associated with 1.92 (IQR: 0.85–2.88, *p* = 0.001) additional HIV cases identified and 1.93 (IQR: 0.86–2.82, *p* = 0.002) more patients initiated on ART each year. Both laboratory and lay health worker cadres were found to have significant non-linearity in their relationships to HIV cases identified each year and patients initiated on ART each year which is graphically displayed in (Additional file 1: Fig. S2). Adjusted models also found borderline significant associations between the pharmacy cadre category and the support staff cadres with both outcomes. The clinical and management categories were not associated with these outcomes in adjusted models.

**Table 3:** Non-parametric regression for effects of healthcare worker category and HIV-program outcomes

	Number of HIV-positive cases identified			Number of HIV-positive cases newly initiated on ART			Viral load suppression rate^1^		
	*β*	(IQR)	*p*	*β*	(IQR)	*p*	*β*	(IQR)	*p*
*Cadre and patient load adjusted models*									
Clinical	− 0.37	(− 0.82, 0.43)	0.798	− 0.36	(− 0.83, 0.46)	0.879	− 0.00	(− 0.02, 0.01)	0.930
Pharmacy	− 3.1	(− 6.42, 1.60)	0.329	− 3.03	(− 5.97, 1.64)	0.358	− 0.05	(− 0.06, 0.01)	0.678
Laboratory	**7.02**	**(3.53, 8.08)**	**0.000**	**6.41**	**(3.31, 7.41)**	**0.000**	− 0.02	(− 0.02, − 0.01)	0.389
Management	− 0.51	(− 1.68, − 0.04)	0.689	− 0.74	(− 1.85, − 0.29)	0.617	0.02	(0.01, 0.05)	0.599
Lay Workers	0.42	(− 0.32, 2.11)	0.284	− 0.36	(− 0.38, 2.09)	0.268	**0.06**	**(0.04, 0.07)**	**0.037**
Support Staff	− 1.07	(− 1.96, − 0.14)	0.323	− 1.21	(− 2.00, − 2.09)	0.255	− 0.03	(− 0.07, − 0.00)	0.411
*Cadre, patient load, region, facility type and health system level adjusted models*									
Clinical	0.04	(− 0.93, 1.00)	0.621	0.04	(− 0.89, 0.95)	0.581	0.06	(− 0.01, 0.12)	0.474
Pharmacy	− 2.08	(− 4.24, − 0.01)	0.082	− 2.03	(− 3.87, − 0.10)	0.091	0.19	(− 0.03, 0.27)	0.500
Laboratory	**3.84**	**(2.00, 7.48)**^**2**^	**0.000**	**3.60**	**(1.74, 6.74)**^**3**^	**0.000**	− 0.03	(− 0.06, 0.01)	0.824
Management	0.12	(− 1.37, 3.27)	0.477	0.10	(− 1.35, 3.21)	0.467	− 0.11	− (0.03, 0.24)	0.657
Lay Workers	**1.92**	**(0.85, 2.88)**^**2**^	**0.001**	**1.93**	**(0.86, 2.82)**^**3**^	**0.002**	0.20	(0.17, 0.28)	0.057^4^
Support Staff	− 1.03	(− 1.83, − 0.10)	0.091	− 0.94	(− 1.71, − 0.05)	0.089	− 0.21	(− 0.31, − 0.13)	0.190

The bold represents values with a *p*-value less than 0.05

*β* Regression Coefficient, *IQR*  25th, 75th centile values

^a^Viral load suppression proportions are expressed as percent in this table

^b^These cadres had non-linear associations with the number of patients identified, the quadratic term coefficients are as follows: Laboratory (*β* = 0.03, IQR:0.02–0.05, *p* < 0.001), Lay worker (*β* = 0.09, IQR:0.05–0.11, *p* < 0.001)

^c^These cadres had non-linear associations with the number of patients initiated on ART, the quadratic term coefficients are as follows: Laboratory (*β* = 0.03, IQR:0.02–0.05, *p* < 0.001), Lay worker (*β* = 0.09, IQR:0.05–0.11, *p* < 0.001)

^d^The lay worker cadre had a non-linear association with viral suppression (*β* = 0.004, IQR:0.003–0.005, *p* = 0.082)

Very little of the observed variability in the proportion of patients achieving viral load suppression was explained by the six staff categories and the number of ART patients being treated at each site (*R*^2^: 0.01). The addition of health region, health system level and type of the facility modestly improved the model’s accuracy (*R*^2^: 0.189), but the majority of inter-site heterogeneity remained unexplained. There was an association between HCWs in the lay worker category, in the crude model, but this relationship was attenuated after confounder adjustment. The adjusted models suggest that addition of one lay worker was associated with a 0.20% (IQR: 0.17–0.28%, *p* = 0.057) increase in the number of patients achieving viral load suppression. This relationship was non-linear, which also displayed in Additional file 1: Fig. S2. No other cadre categories appeared to be associated with viral load suppression rates.

## Discussion

We used data from 216 facilities treating 49,871 HIV-infected patients in Côte d’Ivoire to assess the availability of human resources at HIV prevention, care and treatment facilities and to understand associations between HCW distribution and critical HIV-program outcomes. To our knowledge, this is the first evaluation conducted in SSA to examine the relationship between HCW availability at health facilities and HIV-program outcomes. While more HCWs worked at high ART volume health facilities as compared to ART low volumes facilities, after accounting for patient numbers, we found low volume facilities to have substantially higher staff to patient ratios. This difference was most pronounced in the clinical cadre, where low volume sites had over 2.5-fold higher clinician-to-patient ratios. A previously conducted study in Kenya reported that the maldistribution of clinicians undermined ART service delivery as thousands of HIV-positive individuals especially those in rural areas did not have access to ART [20]. Our analysis demonstrates that policy makers could make data driven decisions regarding the allocation of health workers at ART-facilities based on their patient volume.

Our results show that there were proportionately more lay workers in the low volume health facilities. There has been a rapid emergence of a large number of lay workers across ART programs in SSA [21]. This cadre provides support for HIV testing, initiation on ART, adherence counselling and nutrition support, contacting patients via telephone for appointment reminders, and tracking loss to follow-up patients to bring them back to care [22, 23]. We found this cadre to be associated with a greater number of HIV infected individuals identified, and a higher number of these patients being initiated on ART. Our findings are consistent with previous research which have demonstrated that the use of lay workers increases HIV testing results. A study in Malawi reported that the use of HIV Diagnostic Assistants (a cadre of lay health workers) was associated with a 70% increase (from 28% preintervention to 98% postintervention) in the number of individuals tested for HIV and the identification of HIV-positive cases [24]. Our finding appears to validate the investment in this cadre that has been made by donors, such as PEPFAR in the last decade. Like many countries in SSA, the shortage of clinical HCWs at government sites in Côte d’Ivoire has exerted a huge burden on the remaining HCWs who provide HIV-related services to clients. As a result, lay workers have been recruited and trained to provide these essential services at the facility and community level. Côte d’Ivoire is not alone in this effort, data from Zambia suggested that over 70% of HIV testing services are provided by this cadre[25] and similar findings have also been reported in South Africa and Namibia [26, 27].

Task shifting to lay workers has become a cornerstone of decentralization of ART care [28, 29], and is increasingly recommended as a cost-saving measure to scale-up HIV service delivery in countries across SSA [30]. With support from PEPFAR, many countries have recruited and trained lay workers to initiate patients on ART in the facility or community level, monitor patients and support treatment adherence, thereby achieving better treatment outcomes [31]. In fact, a Cochrane review conducted in 2014 found that shifting the responsibility of HIV care from doctors to nurses or lay workers is safe, and may even improve follow-up of patients after ART initiation [32].

We also found the laboratory cadre to be associated with a higher number of HIV-positive cases identified and a higher number of these cases initiated on ART. Access to laboratory services and the capacity of those services to provide timely results is central to the success of the HIV care cascade [33–35]. It seems plausible that investment in onsite laboratory capacity is associated with improvements in the number of HIV cases identified. In addition, with the very high rate of test-positive patients being initiated on ART observed during this evaluation, this relationship was translated to the ART initiation outcome. We did not find the clinical or pharmacy categories to be strongly associated with any of the outcomes. We might have expected these cadres to have greatest impact on the proportion of new cases who were initiated on ART. Despite wide variation in the clinician to patient ratio across sites, we saw universally high rates of ART initiation. This finding suggests that clinicians and pharmacists in the program may have been able to achieve good patient outcomes even when these cadres were relatively understaffed.

We did observe that once initiated on ART, patients at low volume facilities were slightly less likely to achieve viral load suppression. Clinic-level variation in the number of HIV cases identified and the number of patients started on ART was almost entirely explained by the clinics overall patient volume and its human resources. Conversely, there was very little relationship between patient volume and staffing with the proportion of patients achieving viral load suppression. Côte d’Ivoire is a low-prevalence setting, and previous research conducted by HAI found that retention in HIV-care (which is critical in viral load suppression) is determined by stigma; the quality of previous interactions with medical services; and structural barriers, including costs, waiting times, and availability of medicines [36–38]. We had expected that a higher number of health workforce reduce barriers to care at a site, and thus translate into improved viral load suppression proportions. It is unclear why none of the HCW categories was associated with viral load suppression in our data. This finding may suggest that patient-level factors, ART and clinical factors as well as social and behavioral factors are more likely to influence adherence to ART and viral suppression than site-level human resources [39–41]. Overall, it is important to have multidisciplinary care teams within health facilities providing HIV care and treatment services. These teams made up health professionals (e.g., clinicians, nurses, pharmacists, laboratory technicians, etc.) and other lay cadres (e.g., HIV Diagnostic Assistants, Community Counsellors, etc.) should collaborate in the design and implementation of HIV activities in a health facility as this may contribute to positive results.

The findings of this analysis should be interpreted bearing in mind a few limitations. First, because of the observational design of this evaluation, cause-and-effect relationships cannot be established. Second, human resource categories aggregated multiple types of healthcare workers; therefore, we cannot differentiate specific health personnel (e.g., doctors, midwives, nurses etc.) and their contribution to HIV-program outcomes. Future evaluations should disaggregate the health worker personnel. Third, each category was assessed at a single timepoint, which may misrepresent fluctuations in resources across time. We also cannot account for cadres cross covering each other’s tasks, for example lay workers may shoulder clinical or managerial responsibilities, where there are deficits in those cadres. We aligned our outcome measures with PEPFAR performance metrics critical for achieving the UNAIDs 95-95-95 target. These outcomes cannot be exclusively charactered as either metrics of healthcare access or healthcare quality, but are a product of both access to service and quality of care. Finally, we may have unaccounted for external confounding factors (e.g., incentives, training, quality of services, equipment, medical supplies etc.) which may have contributed to the achievement of the results but were not examined in this analysis. These unmeasured confounders could have attenuated or exaggerated the observed association between HCWs and program outcomes. One important unmeasured confounder is the proportion of HCWs employed by the MOH vs PEPFAR partners, the latter group may be incentivized to focus more on the care of people living with HIV, which may have amplified the association between PEPFAR cadre levels and outcomes.

## Conclusions

We identified marked discrepancies in human resource availability and ART patient volumes at HIV treatment facilities, demonstrating that programmatic data can be used by PEPFAR implementing partners and Ministries of Health to identify gaps in staff availability and make data-driven decision on future human resource investment. Our findings also support investment in laboratory staff and lay worker cadres, which we found to be associated with an increased number of individuals identified as HIV-positive and initiated on ART. It is hard to draw causal inference in this relationship, but our data suggests that the addition of workers in these cadres may prove to be an effective intervention for facilities or regions hoping to improve programmatic performance in these areas. Our findings also suggest that investments to sustain the recruitment, training and proper deployment of HCWs by policy makers at health facilities remain paramount for sustainable HIV epidemic control.

## Supplementary Information

**Additional file 1: Table S1.** Cadre Definitions. **Figure S1.** Number of HCW per category by ART patient volume. **Figure S2.** Lowess smoothed graphs of modelled non-linear relationships between cadre levels and key program outcomes.

## Data Availability

The data sets used and/or analysed during the current study are available from the corresponding author on reasonable request.

## Abbreviations

AIDS: Acquired immune deficiency syndrome
ART: Anti-retroviral therapy
CDC: Centers for Disease Control and Prevention
FBO: Faith-Based Organisation
HAI: Health Alliance International
HCW: Health care worker
HIV: Human immuno virus
NGO: Non-governmental Organisation
PEPFAR: US President’s Emergency Plan for AIDS Relief
SSA: Sub-Saharan Africa
UNAIDS: Joint United Nations Programme on HIV/AIDS
WHO: World Health Organisation
