# Supplementary material for: The association between health workforce availability and HIV-program outcomes in Côte d’Ivoire
Source: Hum Resour Health. 2022 Feb 19;20:18. doi: 10.1186/s12960-022-00715-2 (PMC8858454; doi:10.1186/s12960-022-00715-2)
Supplement: Supplementary file 1 — Additional file 1: Table S1. Cadre Definitions. Figure S1. Number of HCW per category by ART patient volume. Figure S2. Lowess smoothed graphs of modelled non-linear relationships between cadre levels and key program outcomes. [file 12960_2022_715_MOESM1_ESM.docx]

**Additional file 1**

**Table S1: Cadre Definitions**

| **HRH Categories** | **Definition** | **Examples** |
| --- | --- | --- |
| **Clinical** | HCWs who provide direct clinical services to patients | Doctors, nurses, midwives |
| **Pharmacy** | HCWs who dispense ARV at the facility; do not provide direct services to patients | Pharmacy technicians, pharmacy managers |
| **Laboratory** | HCWs who conduct laboratory tests, collect blood samples and relay results to clinicians for diagnostic purposes | Laboratory technicians, laboratorians |
| **Management** | HCWs who provide support to the health facility for administrative needs, but do not provide services to patients | Human resource managers, finance officers, data entry clerks, HIV coordinators, HIV supervisors |
| **Lay Workers** | HCWs who have non-clinical training, but provide direct services to patients | Adherence workers, Community counselors, HIV Diagnostic Assistants and Social Workers. |
| **Support Staff** | HCWs who do not fit into any of the categories above but support the facility in performing HIV activities | Support staff, interns, and volunteers, security guards, cleaners etc |
|  |  | |

**Adapted from PEPFAR Monitoring, Evaluation and Reporting: Indicator Reference Guide, September 2018.**

**Fig. S1: Number of HCW per category by ART patient volume**

**
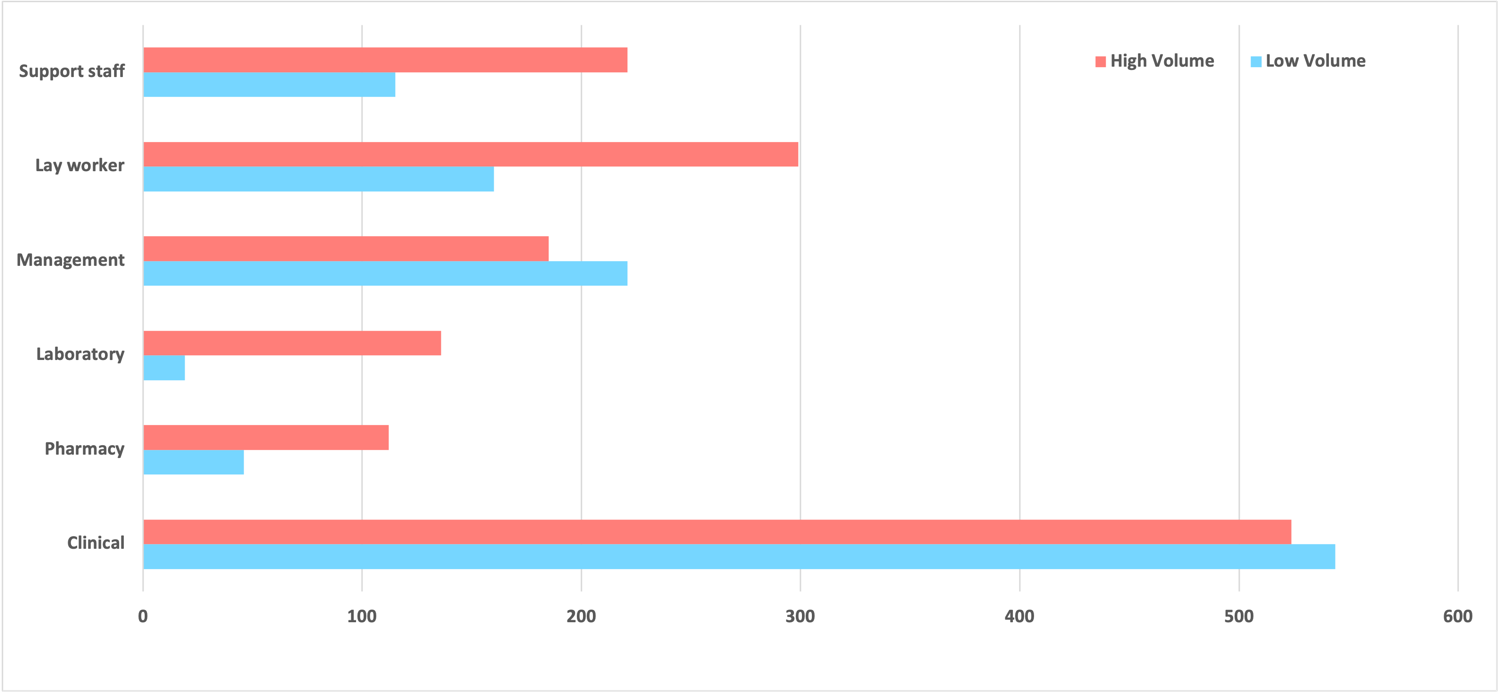
**

**Figure S2: Lowess smoothed graphs of modelled non-linear relationships between cadre levels and key program outcomes.**

E

**
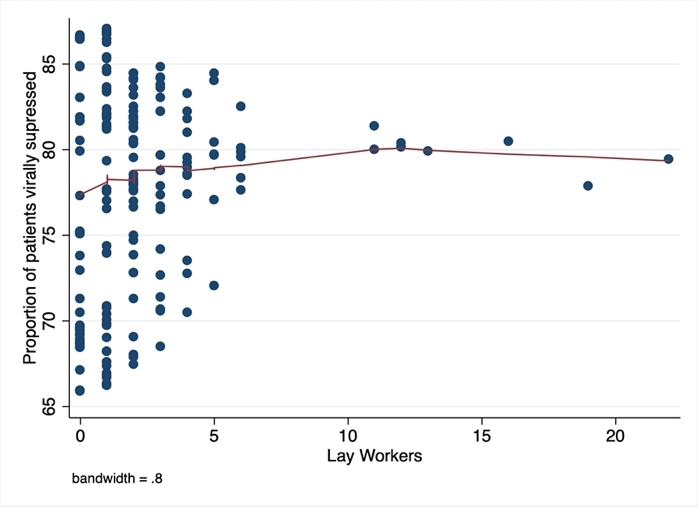
**

A: Adjusted relationship between number of laboratory cadre members at a site and models predicted the number of new HIV cases identified. B: Adjusted relationship between number of lay workers at a site and models predicted the number of new HIV cases identified. C: Adjusted relationship between number of laboratory cadre members at a site and models predicted the number of new HIV cases initiated on ART. D: Adjusted relationship between number of lay workers at a site and models predicted the number of new HIV cases initiated on ART. E: Adjusted relationship between number of lay workers at a site and models predicted percentage of patients achieving viral load suppression. All models are adjusted for the number of clinicians, pharmacists, managers, patient in treatment at the start of the observation period, region, facility type, and facility classification.
